# Abnormal Striatal BOLD Responses to Reward Anticipation and Reward Delivery in ADHD

**DOI:** 10.1371/journal.pone.0089129

**Published:** 2014-02-26

**Authors:** Emi Furukawa, Patricia Bado, Gail Tripp, Paulo Mattos, Jeff R. Wickens, Ivanei E. Bramati, Brent Alsop, Fernanda Meireles Ferreira, Debora Lima, Fernanda Tovar-Moll, Joseph A. Sergeant, Jorge Moll

**Affiliations:** 1 Okinawa Institute of Science and Technology Graduate University (OIST), Okinawa, Japan; 2 D’Or Institute for Research and Education (IDOR), Rio de Janeiro, Brazil; 3 Federal University of Rio de Janeiro, Rio de Janeiro, Brazil; 4 University of Otago, Dunedin, New Zealand; 5 VU University Amsterdam, Amsterdam, The Netherlands; Chiba University Center for Forensic Mental Health, Japan

## Abstract

Altered reward processing has been proposed to contribute to the symptoms of attention deficit hyperactivity disorder (ADHD). The neurobiological mechanism underlying this alteration remains unclear. We hypothesize that the transfer of dopamine release from reward to reward-predicting cues, as normally observed in animal studies, may be deficient in ADHD. Functional magnetic resonance imaging (fMRI) was used to investigate striatal responses to reward-predicting cues and reward delivery in a classical conditioning paradigm. Data from 14 high-functioning and stimulant-naïve young adults with elevated lifetime symptoms of ADHD (8 males, 6 females) and 15 well-matched controls (8 males, 7 females) were included in the analyses. During reward anticipation, increased blood-oxygen-level-dependent (BOLD) responses in the right ventral and left dorsal striatum were observed in controls, but not in the ADHD group. The opposite pattern was observed in response to reward delivery; the ADHD group demonstrated significantly greater BOLD responses in the ventral striatum bilaterally and the left dorsal striatum relative to controls. In the ADHD group, the number of current hyperactivity/impulsivity symptoms was inversely related to ventral striatal responses during reward anticipation and positively associated with responses to reward. The BOLD response patterns observed in the striatum are consistent with impaired predictive dopamine signaling in ADHD, which may explain altered reward-contingent behaviors and symptoms of ADHD.

## Introduction

Attention deficit hyperactivity disorder (ADHD) is characterized by elevated levels of inattention, overactivity and/or impulsivity that impair daily functioning. The disorder is common and demonstrates continuity across the life span affecting 5.9–7.1% of children and 5% of adults [1]. The etiology of ADHD is not completely understood. Genetic factors play a role but probably involve multiple genes of moderate effect rather than a single gene. Associations have been established with genetic variations in the dopamine D4 receptor [2], [3] and the dopamine transporter (DAT1) [4], the dopamine D5 receptors, serotonin transporters [5] and dopamine beta-hydroxylase (DBH), 5-hydroxytryptamine (serotonin) receptor 1B (HTR1B) and synaptosomal-associated protein, 25 kDa (SNAP-25) genes [6], [7]. A reduction in dopamine synaptic markers including DAT1 has also been shown in the dopamine reward pathway [8]. Altered dopamine function may lead to changes in reward mechanisms in people with ADHD [9]–[12].

Consistent with genetic mechanisms affecting reward pathways, a number of experimental studies suggest children with ADHD differ from typically developing children in their responses to reward [13]. This has been measured by effects on cognitive task performance [14]–[18], psychophysiological variables [16], [19]–[22] and choice behavior [23]–[32]. Although there is considerable variability across these studies, a consistent finding in choice behavior is a stronger preference for small immediate rewards over larger delayed rewards [33]. This has been shown in temporal discounting [24], [25], choice delay tasks [23], [26]–[30] and signal detection procedures [32]. Recent studies with adults with ADHD indicate a similar preference for, or sensitivity to, immediate versus delayed reward compared to controls [34], [35].

The pathophysiological mechanism underlying this behavioral sensitivity to delay of reinforcement in individuals with ADHD is, however, unclear. Normally, when reward is delayed, cues that predict reward can bridge the delay by acting as conditioned reinforcers [36]–[38]. The neural mechanism underlying this bridging effect may involve the transfer of dopamine cell responses from established reinforcers to cues after repeated pairings, as shown in animal studies [39]–[42]. This transfer of the dopamine cell firing to earlier cues predicting reinforcement may explain how behavior can be maintained in situations where external reinforcement is delayed.

While a number of brain regions contribute to producing the dopamine responses to reward cues and receipt of reward, the output of the dopamine cells is highly concentrated in the striatum, which receives 100-fold higher density of dopamine innervation than other brain regions [43], [44]. Consistent with this, functional magnetic resonance imaging (fMRI) studies in humans have shown blood-oxygen-level-dependent (BOLD) responses in the ventral striatum during the anticipation of primary and secondary rewards [45], [46] after conditioning. These BOLD responses have been shown to correlate positively with positron emission tomography measures of dopamine release in the striatum [47]. Pharmacological evidence also suggests that dopamine release, by activating postsynaptic D1 receptors in the striatum, leads to increased BOLD signals [48].

We hypothesize that in ADHD, the transfer of the phasic dopamine release from reward to reward-predicting cues is deficient [12]. Previous studies have shown reduced BOLD activity during reward anticipation, consistent with hypodopaminergic function [49]–[53], and negative associations between such activation and ADHD symptoms (also [54], but not [51]). On the other hand, Paloyelis et al. [55] reported increased responses to reward receipt in the dorsal striatum (caudate nucleus) of adolescents with ADHD. These results are consistent with our hypothesis but do not exclude other possible explanations such as overall hypodopaminergic or hyperdopaminergic function. To specifically test our hypothesis, we studied both anticipatory responses and responses to reward delivery in a simple classical conditioning paradigm similar to those used in the animal studies that first showed the transfer of dopamine cell responses from reward to reward-predicting stimuli [56].

We examined fMRI signals in young adults with lifetime symptoms of ADHD and well-matched controls, as *indirect* measures of dopamine release. We predicted that control participants would demonstrate increased striatal BOLD responses to reward cues together with decreased responses to reward delivery as previously demonstrated (e.g., [57]). Conversely, we predicted reduced BOLD responses to reward cues, but stronger responses to reward delivery in the ADHD group, possibly due to a failure to establish phasic dopamine responses to reward-predicting cues. Alternatively, the hypodopaminergic hypothesis would predict an overall reduction in dopamine signaling [11], and thus reduced responses to both reward cue and reward delivery in those with ADHD symptoms compared to controls.

## Materials and Methods

### *Ethics Statement*

The study was approved by the ethics committees of the Institute of Psychiatry of the Federal University of Rio de Janeiro (Brazil) and the D’Or Institute for Research and Education (Brazil). All participants provided written informed consent and received a gift voucher in the amount earned in the fMRI task (amounts varied between R$129 and R$141).

### *Participants*

Participants were recruited from amongst medical students attending the 9^th^ semester of their course at the Federal University of Rio de Janeiro, Brazil, between 2008 and 2011. All participants belonged to upper-middle and upper socioeconomic classes (Class A and B according to IBGE, Brazilian socioeconomic classification, www.ibge.gov.br). Three hundred and ninety seven students completed a screening measure of ADHD symptoms (Portuguese version of the Adult Self Report Scale, [58]). Students who endorsed more than 4 current symptoms of inattention *and/or* hyperactivity/impulsivity were classified as potentially eligible for the ADHD group, and those who endorsed less than 3 current symptoms were classified as potential controls. They were invited to participate in a comprehensive assessment of past and current symptoms of ADHD and comorbidity, by qualified psychiatrists, trained and supervised by a senior psychiatrist/researcher (PM), who also reviewed all diagnostic decisions. Semi-structured interviews were administered to confirm the presence and severity of the ADHD symptoms, or their absence (for controls), and to evaluate disorders with high rates of comorbidity with ADHD in adults. The psychiatrists also reviewed other psychological and medical conditions with the students during the interview.

The Attention Deficit Hyperactivity Disorder module of the Portuguese version of the Kiddie-Schedule for Affective Disorders and Schizophrenia-PL (KSADS-PL) [59] was used to assess 18 symptoms of ADHD listed in the DSM-IV and 5, and only symptoms causing impairments were considered to be present. Comorbidity (Mood Disorders, Anxiety Disorders, Substance-Related Disorders, Psychotic Disorders and Eating Disorders) was assessed using the Mini-International Neuropsychiatric Interview in Portuguese (M.I.N.I.) [60]. The inter-rater reliabilities of the original (.64–.67) [61] and Portuguese (.91–1.00, with adults) versions of the KSADS-PL ADHD section [59] as well as of the M.I.N.I. (.88–1.00), and its agreement with the Composite International Diagnostic Interview (CIDI) (.36–.82) and Structured Clinical Interview (SCID) (.51–.84) [62] have been reported to be adequate.

The eligibility criteria for the fMRI study were: no current drug use, psychotic symptoms, major depression or anxiety disorder; no history of neurological disorder; right-hand dominant [63]; and no MRI contraindications. Eighteen students (10 males, 8 females), meeting DSM-IV diagnostic criteria for ADHD in childhood and demonstrating at least 5 current symptoms of inattention and/or hyperactivity/impulsivity formed the ADHD group. The DSM-5 criteria for the predominantly inattentive presentation based on current symptoms were met by 4 participants, hyperactive/impulsive presentation by 3 participants, and combined presentation by 11 participants. Sixteen students (8 males, 8 females) demonstrating fewer than 3 current or previous symptoms of ADHD, meeting the study eligibility criteria and matched for gender and age, took part as control participants. All participants were medication-naïve for stimulant medications.

One student from the control group and 4 students from the ADHD group were excluded from the final fMRI analyses due to movement-related or other artifacts. The final sample included 14 participants in the ADHD group (8 males, 6 females) and 15 participants in the control group (8 males, 7 females). The mean age (ADHD = 23 years, sd = 1.41; control = 23 years, sd = 1.41) and estimated IQ (ADHD = 113, sd = 8.57; control = 111.53, sd = 7.49; Wechsler Abbreviated Scale of Intelligence, [64]) of the two groups were similar. There were no significant differences in the head motion (3 translation, 3 rotation) parameters between the ADHD (mean = 0.18, max = 2.25) and control (mean = 0.22, max = 2.96) groups [65]. Additionally, no significant stimulus-correlated motions were observed (*r* <0.2) in either group (http://www.nitrc.org/projects/artifact_detect/) [66].

### *fMRI Paradigm*

**Figure 1:**
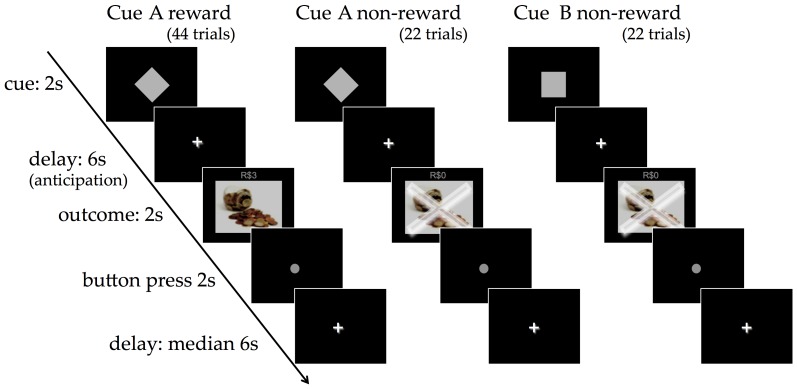
Classical conditioning fMRI paradigm. One of two neutral stimuli (Cue A or Cue B) was followed by an outcome stimulus (reward or non-reward) after a 6-second delay. Cue A was followed by the delivery of the reward 66% of the time and non-reward 33% of the time. Cue B was always followed by non-reward. Participants were told they would receive the equivalent of R$3 for each reward outcome. The length of the inter-trial delay was varied.

A classical conditioning paradigm was developed to examine BOLD responses to reward anticipation and delivery, unconfounded by operant behavioral responses, decision-making or complex reward probability and magnitude estimation (Figure 1). One of two initially neutral stimuli (Cue A or Cue B) was followed by a 6 second delay (anticipation period) after which an outcome stimulus (reward or non-reward) was presented. The delay was kept constant to maintain the temporal predictability of the outcome delivery. A picture of coins was presented as a reward outcome and the participants were told they would receive the equivalent of R$3 for each reward outcome and nothing for the non-reward outcomes. Cue A was followed by reward 66% of the time (44 trials) and non-reward 33% of the time (22 trials) to allow a level of uncertainty. Cue B was always followed by non-reward (22 trials). Participants were asked to press a button when a target appeared after the outcome (Lumina, Cedrus Corporation, CA). The length of the inter-trial delay was varied to reduce the temporal predictability of the next trial (Poisson distribution: min = 4.5 s, max = 14.5 s, average = 6.5 s, sd = 2.05 s).

### *fMRI Image Acquisition*

Participants were scanned in the afternoon, except for 1 ADHD and 2 control participants scanned in the morning. Prior to scanning, participants were given brief instructions followed by a practice using a PowerPoint presentation and a computer key, during which all possible conditions/outcomes of the fMRI paradigm were presented once. Extended training and testing of an explicit awareness of the reward contingency were avoided to discourage cognitive coding of the cues and solicited attention to the cues over reward, as instructed knowledge [67] and extended training [68] have been documented to alter striatal responses. Participants were told to pay attention to two symbols (square or diamond) and subsequent outcomes (reward or non-reward), and to press a button at the end of the trial to indicate their attention to the images. It was emphasized that money awarded in the scanner would be converted to bookstore gift vouchers.

Functional images were acquired in two runs with a 3T Achieva scanner (Philips Medical Systems, The Netherlands). Each run consisted of 44 trials (22 Cue A reward, 11 Cue A non-reward, 11 Cue B non-reward trials), presented in a pseudo-randomized order, using an event-related design. The functional scanning time was 852.5 seconds for the first run and 840.5 seconds for the second run (the difference is due to the varied inter-trial delay periods), for a total of 29 minutes. This yielded 426 functional images for the first run and 420 images for the second run. The trial number, sequence and time were uniform across all the participants. Task stimuli were shown using an LCD display and a mirror adapted to the head coil. Foam padding and straps over the forehead and under the chin were used to restrict head motions.

An 8-channel SENSE head coil, single-shot T2*-weighted fast-field echo, echo-planar imaging sequence (TR = 2000 ms, TE = 22 ms, Matrix = 80×80, FOV = 240 mm, flip angle = 90°, 3 mm isotropic voxel size, 36 slices in ascending order with no gap), a SENSE factor of 2, and “dynamic stabilization” were used to optimize the acquisition of the signals especially in the subcortical areas [69], [70]. Reference anatomical images were acquired using a T1-weighted 3D magnetization-prepared, rapidly acquired gradient echo sequence (TR/TE = 7.2/3.4 s, Matrix/FOV = 240/240 mm, flip angle = 8°, 1 mm isotropic voxel size, 170 sagittal slices).

### *fMRI Analysis*

fMRI data were analyzed with SPM8 (www.fil.ion.ucl.ac.uk/spm/software/spm8, in MATLAB R2009b, www.mathworks.com) employing the general linear model [71], [72]. All trials were included in the analysis as we expected learning at the neural level to be accomplished quickly given the simplicity of the task.

Preprocessing was completed using realignment, slice-time correction (referencing middle slice), co-registration, and normalization to the standard MNI EPI template (Montreal Neurological Institute brain template), resulting in the reconstructed functional images with voxel dimensions of 3 mm^3^. Images were spatially smoothed using a 6 mm full-width half-maximum Gaussian spatial kernel to optimize the detection of subcortical BOLD signals [73]. Unwanted low frequencies were removed using high-pass filtering (128 s) [74]. Nine condition-specific regressors (Cue A, Cue B, Cue A delay, Cue B delay, Cue A reward, Cue A non-reward, Cue B non-reward, Button press and Fixation) were convolved with the canonical hemodynamic response function for each participant [75]. Contrasts were performed to examine BOLD responses specific to reward anticipation (Cue A delay vs. Cue B delay) and to reward delivery (Cue A reward vs. Cue B non-reward) while controlling for all other conditions in the first-level analysis. The resulting contrast images were entered into the second-level, one-sample t-tests for within-group analysis and two-sample t-tests for between-group analysis with a random-effects model.

The alpha level to identify significant fMRI responses was set to *p*<.05, using family-wise error (FWE) corrections for multiple comparisons with a minimum cluster size of 5 voxels. A whole-brain analysis was conducted first, and *a priori* defined regions of interest (ROIs) were examined, using small volume correction (SVC). Striatal regions consistent with the previous studies of reward sensitivity in ADHD [49]–[53], [55], [76]–[78] were examined for the contrasts of interest. MNI coordinates (x, y, z) for each *a priori* ROI were derived from a meta-analysis [79] (ventral striatum [nucleus accumbens]: 12/−12, 10, −6, dorsal striatum [caudate]: 8/−8, 14, 2). For SVC, anatomical ROIs were created in MRIcroN (http://www.mccauslandcenter.sc.edu/mricro/mricron/) by specifying spheres (radius = 10 mm) centered around these *a priori* coordinates, including only gray matter (GM) structures (based on T1-based GM templates) as to discard structures containing mostly cerebrospinal fluid or white matter voxels.

Mean beta values within each anatomical ROI, extracted for each contrast of interest for each participant, were used to examine the group (control vs. ADHD) and condition (reward anticipation vs. delivery) interaction effects in the ventral and dorsal striatum (IBM SPSS Statistics version 20). To examine the relationships between BOLD responses and ADHD symptoms, the number of current inattention and hyperactivity/impulsivity symptoms was modeled separately as a covariate at the second-level SPM analysis (one-sample t-test) for the reward anticipation and outcome in the ADHD group.

Data is available at the BrainMap (www.brainmap.org, searchable by the publication information such as the author, journal name, year of publication, key words).

## Results

Analyses focused on striatal responses to reward anticipation and reward delivery in the ADHD and control groups. We predicted that the ADHD group would demonstrate reduced BOLD signals during reward anticipation but greater activation in response to reward delivery, compared to the controls.

### *Responses to Reward Anticipation*

**Figure 2:**
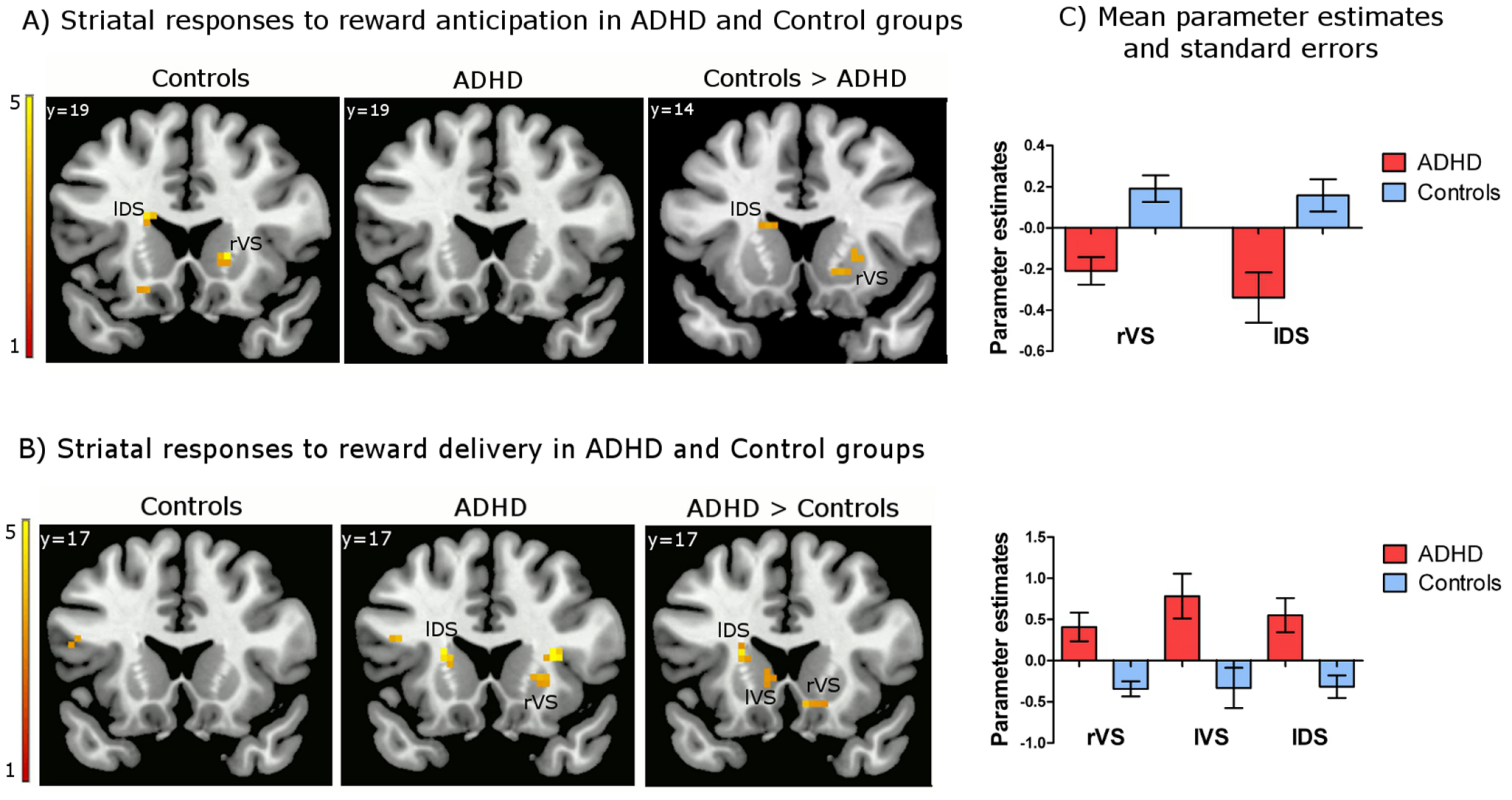
Striatal responses to reward anticipation and reward delivery in the ADHD and control groups. Brain activation displays were generated by overlaying SPM t-maps resulting from the group-level analyses on an MNI standard brain (*p*<.005 uncorrected, cluster size ≥5 voxel, for visualization purposes) and applying a gray matter mask. (A) Increased activation in the left dorsal striatum (head of caudate) and right ventral striatum (nucleus accumbens and ventral putamen) during reward anticipation in the control group. (B) Increased activation in the left dorsal striatum (head of caudate), left ventral striatum (ventral regions of the head of caudate), and right ventral striatum (nucleus accumbens and ventral putamen) in response to reward delivery in ADHD. (C) Bar graphs represent mean parameter estimates and standard errors from the GLM analyses examining the effects of reward anticipation (Cue A delay – Cue B delay contrast) and reward delivery (Cue A reward – Cue B non-reward contrast), which were extracted from the local maxima observed within the *a priori*-defined ROIs based on a meta-analysis (Liu et al., 2011); MNI x, y, z = 18, 17, −5 for rVS, −15, 8, 16 for lDS in response to reward anticipation; MNI x, y, z = 9, 17, −11 for rVS, −9, 17, 1 for lVS, −18, 8, 16 for lDS in response to reward delivery. These graphs are provided for illustrative purposes only, and were not used for statistical inferences.

Within-group analyses revealed increased BOLD responses in right ventral striatum (rVS) and left dorsal striatum (lDS) in the control group during reward anticipation (Cue A delay – Cue B delay) (Figure 2, Table 1). In contrast, consistent with our predictions, there were no statistically significant hemodynamic effects during reward anticipation in the ADHD group. Between-group comparisons (Control – ADHD) indicated significantly greater responses in rVS and lDS in the controls, compared to the ADHD group.

**Table 1:** BOLD responses to reward anticipation and reward delivery in the ADHD and control groups.

Anatomical region	Side	Clustersize	MNI coordinates(x, y, z)			Z-score
**Reward anticipation (Cue A delay vs. Cue B delay)**						
***Control***						
Ventral striatum	R	11	18	20	1	3.76*
Dorsal striatum	L	6	−18	20	19	3.50*
***ADHD***						
No significant activation						
***Control>ADHD***						
Ventral striatum	R	26	18	17	−5	3.71**
Dorsal striatum	L	24	−15	8	16	3.13**
***ADHD>Control***						
No significant activation						
**Reward delivery (A reward vs. B non-reward)**						
***Control***						
No significant activation						
***ADHD***						
Putamen	R	8	24	14	1	2.99*
Dorsal striatum	L	27	−21	17	13	4.09*
***Control>ADHD***						
No significant activation						
***ADHD>Control***						
Ventral striatum	R	21	9	17	−11	3.31**
Ventral striatum	L	7	−9	17	1	2.79*
Dorsal striatum	L	18	−18	8	16	3.11**

Notes: Coordinates are for the significant local maxima of clusters in the random effects analyses (MNI: Montreal Neurological Institute coordinate system). T-statistics were converted to z scores. For the effects surviving SVC, corrected z scores are reported.

*Trend-wise significant, *p* = .005 uncorrected, *k* ≥5;

**Corrected for small volume, *p* = .05 FWE-corrected.

### *Responses to Reward Delivery*

No significant increase in BOLD activation in the ventral or dorsal striatum was observed in the control group in response to reward delivery (Cue A reward – Cue B non-reward) (Figure 2, Table 1). In contrast, increased responses were observed in the right putamen and left dorsal striatum in the ADHD group. When using a more lenient statistical threshold (*p*<.01, uncorrected), a response in the orbitofrontal cortex was also observed in the ADHD group. However, this was not significant after FWE corrections or SVC. Between-group analysis (ADHD – Control) identified greater hemodynamic effects in bilateral VS and lDS in ADHD participants in response to reward delivery, relative to controls. An additional comparison (Cue A reward – Cue A non-reward) did not show statistically significant effects.

### *Group×Condition Interaction*

Analyses of variance (mixed-design) were conducted to confirm the group (ADHD vs. Control)×condition (reward anticipation vs. delivery) effects. Significant interaction effects for the responses in bilateral VS and lDS and a main effect of the condition for lDS were observed (Table 2).

**Table 2:** The interaction effects of the group (control vs. ADHD) and condition (reward anticipation vs. reward delivery).

Factor	Mean (std. error)				F	*p*	Partial eta^2^
**Right ventral striatum**							
Group	Control	−.070 (.073)	ADHD	.029 (.076)	.884	.*355*	.032
Condition	Anticipation	.024 (.039)	Delivery	−.066 (.112)	.475	.*496*	.017
Group**x**Condition	Control/Anticipation	.091 (.054)	Control/Delivery	−.232 (.156)	3.165†	.*086*	.105
	ADHD/Anticipation	−.043 (.056)	ADHD/Delivery	.100 (.161)			
**Left ventral striatum**							
Group	Control	−.104 (.077)	ADHD	.083 (.080)	2.803	.*106*	.094
Condition	Anticipation	−.004 (.045)	Delivery	−.016 (.106)	.010	.*921*	.000
Group**x**Condition	Control/Anticipation	.076 (.062)	Control/Delivery	−.284 (.148)	8.526**	.*007*	.240
	ADHD/Anticipation	−.085 (.064)	ADHD/Delivery	.251 (.153)			
**Left dorsal striatum**							
Group	Control	−.024 (.053)	ADHD	.054 (.054)	1.051	.*314*	.037
Condition	Anticipation	−.099 (.039)	Delivery	.130 (.083)	4.689*	.*039*	.148
Group**x**Condition	Control/Anticipation	.001 (.054)	Control/Delivery	−.048 (.116)	6.934*	.*014*	.204
	ADHD/Anticipation	−.200 (.056)	ADHD/Delivery	.308 (.120)			

Note. df = 1, 27;

† *p*<.10,

*p<.05,

**p<.01.

### *Relationship between BOLD Responses and ADHD Symptoms*

**Figure 3:**
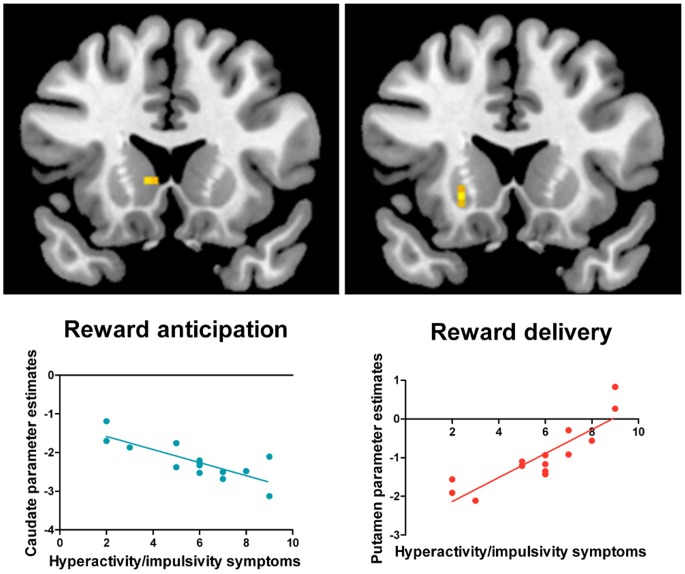
Association between ventral striatal responses and the number of hyperactivity/impulsivity symptoms in the ADHD group. Brain activation displays were generated by overlaying SPM t-maps from the second-level analyses on a MNI standard brain (*p*<.005 uncorrected). Reward anticipation (Cue A delay), t = 4.25 (*p*<.005, uncorrected, MNI local maxima: x, y, z = −6, 14, 4); reward delivery (Cue A reward), t = 5.43 (*p* = .04, FWE corrected, MNI local maxima: x, y, z = −21, 20, −5). The graphs depict the association between the mean parameter estimates of the peak cluster from the one-sample t-tests (y-axis) and the number of symptoms (x-axis) for each participant in the ADHD group. The graphs are provided for illustrative purposes only, and were not used for statistical inferences. MNI: Montreal Neurological Institute.

Dimensional analyses were conducted for the ADHD group by modeling the number of symptoms as a predictor variable and BOLD responses to reward anticipation (*Cue A delay*) and reward delivery (*Cue A reward*) as dependent variables, and other conditions as control variables, using within-group one-sample t-tests, SPM second-level analysis. The relationships between the individuals’ responses to each condition (rather than contrasts between two conditions) and the number of ADHD symptoms were examined. A higher number of current symptoms of hyperactivity/impulsivity was associated with weaker BOLD signals in left ventral caudate during reward anticipation, together with stronger signals in left ventral putamen in response to reward delivery (Figure 3). A positive association was also found between the number of current inattention symptoms and bilateral VS activation in response to reward delivery; this effect did not survive FWE corrections.

## Discussion

The current study revealed altered BOLD responses to a reward-predicting cue and reward delivery among high-functioning young adults with elevated lifetime symptoms of ADHD compared to controls. During reward anticipation, no significant striatal responses were observed in the ADHD group, in contrast to the well-matched controls, who demonstrated increased responses in the ventral and dorsal striatum. Conversely, in response to reward delivery, the ADHD group showed significantly greater hemodynamic effects in the ventral and dorsal striatum than the control group. To our knowledge, this is the first demonstration of a reversal of striatal activation in response to a reward cue and reward delivery in individuals with clinical levels of ADHD symptoms, relative to the control group.

The pattern of BOLD responses observed in control participants is consistent with dopamine release in response to reward and reward-predicting stimuli recorded in the ventral and dorsal striatum in animal studies. These studies, using similar conditioning paradigms, have shown that the phasic responses of dopamine cells transfer from unexpected primary rewards to reward-predicting stimuli [40], [56], [80]. Similarly, in humans, striatal activity has been seen in response to reward-predicting cues [45], [57], [81], [82]. The control participants in the current study showed increased BOLD responses in the striatum during reward anticipation, but very little activity in response to reward delivery, implying that a transfer of phasic responses has occurred.

In contrast to controls, the ADHD group showed the reverse pattern, with no significant increase in striatal responses during reward anticipation but increased responses to reward delivery. This finding is consistent with the hypothesis of an impaired transfer of dopamine cell firing from reward to reward-predicting cues in ADHD [12]. These data are not consistent with overall dopamine hypofunctioning [11], which would predict decreased responses to both reward cues and reward delivery.

The present study is the first to report ADHD versus control group differences in BOLD responses to *both* reward anticipation and delivery within the same study. Previous studies have shown either that cue-evoked BOLD responses are lower among individuals with ADHD [49]–[53], or that striatal activation is increased in response to reward delivery [55]. We report a reciprocal dissociation that may only have been detected because the current study was designed to test the dopamine transfer deficit hypothesis. We used a simple classical conditioning task modeled on animal studies tailored to measure phasic dopamine release to predictive cues and reward. The paradigms used in other fMRI studies [49]–[53], [55], [78], [83] differ in important details, such as having a requirement for a behavioral response before reward delivery.

Participants in both the ADHD and control groups in this study were recruited from a population of medication-naïve, high-functioning young adults. This resulted in homogenous groups that differed on the variable of interest only, symptoms of ADHD. While the participants in the ADHD group had not been diagnosed previously, the semi-structured interview revealed clinical levels of lifetime ADHD symptoms, severe enough for a diagnosis. It could be argued that untreated high-functioning adults with elevated ADHD symptoms represent a small portion of affected individuals [84] and that a previously diagnosed population would have more severe symptoms and functional impairments. Therefore, in comparing our results with those from previously diagnosed adults, some allowance should be made for the possibility of more marked changes in the diagnosed and less marked changes in the non-diagnosed adults due to biological adaptation or the effects of medication. Our findings may not generalize to other clinical populations with greater functional impairments. However, the current finding of the reduced VS responses during reward anticipation in the ADHD group is consistent with previous reports in clinical samples [49]–[53].

The dorsal striatum also showed increased BOLD responses during reward anticipation in controls, and to reward delivery in the ADHD group. Such altered dorsal striatal activation in ADHD samples has not been shown previously, but is consistent with previous research suggesting that individuals with ADHD may value the receipt of reward more highly than its anticipation [83]. At a mechanistic level, animal studies show that the dorsal striatum exhibits neural activity in relation to the learned association of sensory cues with movements [85] and like the ventral striatum [86], receives a strong dopaminergic reward signal [44]. Therefore, we speculate that the dorsal striatum responses in the present study could be related to the dopamine action on striatal neurons becoming active during preparation for the button-press movement required after reward delivery.

The present study focused on BOLD signals in the dorsal and ventral striatum because the highest density of dopamine innervation is found in these areas and our hypotheses were derived from possible underlying mechanisms of dopamine action in ADHD. It should be emphasized that many different brain regions outside of the scope of the present study also contribute to the processing of reward and may be important in the pathophysiology of ADHD. The importance of the dynamic relationships between different brain regions and functions in defining altered reward sensitivity is increasingly recognized [87] and may be a goal to be pursued in further studies. For example, it could be argued that the differences we observed were due to an overall lack of attention in the participants with elevated ADHD symptoms, which might have caused reduced BOLD signals in response to cues but not in relation to reward delivery.

The current paradigm was simple enough to allow the neural learning to occur in the first few trials (e.g., [57]). Thus, we are unable to determine whether the transfer of striatal responses to a reward-predicting cue is slower among individuals with ADHD or if their responses converge to a different asymptote. An important extension to the current study would be to investigate the transfer of dopamine-associated BOLD responses over time and the relationship between changes in neural activity and behavioral learning.

In the present study, we focused on the contrast of reward delivery versus signaled non-reward outcome. Another question of interest is whether brain responses to non-reward (expected or unexpected) are altered in adults with ADHD symptoms. Previous studies in animals and healthy human participants found BOLD “deactivations” in the striatum when expected rewards are omitted [88]. Our additional exploratory analyses contrasting reward delivery with unexpected non-reward indicated no significant change in activation in the ADHD versus control group. In exploring effects of reward omission, the contrast of unexpected versus signaled non-reward suggested greater striatal responses to unexpected non-reward in the ADHD group. The control group showed little activation in response to either expected or unexpected non-reward. Although beyond the scope of the current study, developing predictions and systematically examining the responses to unexpected versus signaled non-reward in ADHD versus healthy controls would provide further insight into how the behaviors of individuals with ADHD are affected by reward and its omission.

Previous studies with ADHD clinical samples have described an inverse relationship between the striatal responses during reward anticipation and hyperactivity/impulsivity symptoms [49], [50], [52]. In contrast, impulsivity traits have been found to positively correlate with anticipatory striatal responses in healthy adolescents and adults [89]. Our results are consistent with the earlier studies using clinical populations, also showing a negative correlation between ventral striatal responses during reward anticipation and the number of current hyperactivity/impulsivity symptoms.

We suggest that among adults with clinical levels of ADHD symptoms, there may be a failure of anticipatory dopamine release in response to reward-predicting cues, which would result in a lack of continuous cellular reinforcement in the striatum when behavioral reward is delayed or discontinuous. Individuals with ADHD may therefore engage in behaviors that provide them with immediate and continuous external reward, which manifest as symptoms of ADHD.

Conversely, in the current study, striatal activation in response to reward delivery was positively associated with current hyperactivity/impulsivity and inattention symptoms. We suggest that in ADHD, there is continued firing of dopamine neurons in response to actual rewards. This would result in reinforcement of “off-task” or alternative behaviors, leading to symptoms of inattention and hyperactivity/impulsivity [12].

Our fMRI results, together with findings from animal studies, suggest a biological basis for abnormal sensitivity to reinforcement in ADHD. The failure of anticipatory dopamine would explain some of the behavioral results reported in the literature including: a stronger preference for immediate over delayed reward [32], [90], poorer performance when reward is not continuous [91], and the need for consistent and immediate reward for shaping behavior [92] amongst those with ADHD. Failure to anticipate reward coupled with continued responses to actual rewards may also offer a biological explanation for the delay aversion observed in children [23], [26]–[30] and adults [34], [35] with ADHD. For typically developing individuals, reward anticipation serves to maintain appropriate behavior during the periods of waiting. For those with ADHD, the symptoms of inattention, hyperactivity and impulsivity may arise from behaving in ways that lead to immediate reinforcement.

As noted above, the current study is not without limitations. 1) While our high-functioning adult sample displayed elevated levels of lifetime symptoms of ADHD, they had not received a diagnosis of ADHD prior to the clinical interview conducted for the study, thus the generalizability of the current results to the populations with more severe symptoms and functional disabilities may be limited. 2) Our sample size was relatively small, which may have limited statistical power to detect BOLD responses in other areas of brain in the whole-brain analysis. This also prevented us from examining possible interactions with other structures of the reward circuitry. 3) Three of the participants were assessed in the morning, while all others were assessed in the afternoon, possibly affecting their reward-related responses. 4) The present findings do not exclude the possibility that the observed pattern of striatal responses to reward anticipation and delivery may not be specific to ADHD and are present in other pathological conditions associated with altered reward sensitivity. 5) Our study does not exclude the possibility that the behavioral symptoms of ADHD may also relate to, or caused by, different neurobiological processes [93]. Nonetheless, the current study represents a significant step in teasing out the possible underlying mechanism of altered reward sensitivity in ADHD, and the findings argue for behavioral and pharmacological interventions that target the impaired transfer of dopamine release from reward to reward predicting cues.
